# Mothering away from home: experiences of Zimbabwean domestic workers in South Africa

**DOI:** 10.3389/fsoc.2025.1607624

**Published:** 2025-09-25

**Authors:** Londeka Zamankwali Shamase, Lusanda Sekaja

**Affiliations:** Department of Industrial Psychology and People Management, University of Johannesburg, Johannesburg, South Africa

**Keywords:** domestic worker, ambiguous loss, transnational mothering, emotional challenges, financial challenges, visitational challenges, Zimbabwe, South Africa

## Abstract

Many women leave their homes in Zimbabwe in the hopes of finding work in South Africa. In this study we investigated the experiences of Zimbabwean transnational mothers working as domestic workers in South Africa with a specific focus on how they navigate motherhood while separated from their children. We conducted semi-structured interviews to explore the visitational, financial, and emotional aspects of their separation. We used thematic analysis to interpret the data, which revealed three key themes, namely, (a) infrequent homecoming shaped by structural barriers, (b) vulnerabilities and unseen challenges of children left behind, and (c) transnational mothering. The findings highlight the emotional tax and structural constraints of mothering across borders. The study calls for a more culturally grounded understanding of parenting, specifically transnational mothering.

## 1 Introduction

According to Statistics South Africa (2024), there are 861,000 domestic workers in South Africa. They make up about 25% of the informal labor sector, agriculture excluded. Although the exact numbers are not known, Zimbabwean domestic workers make up a large portion of this group. This is not surprising, given that globally, one in every five domestic workers is employed in a country outside their own (International Labour Organization., 2021). Owing to South Africa's proximity to Zimbabwe and better prospects of finding work there than in other neighboring nations, the country is often an attractive destination for migrant women seeking work opportunities, including domestic work. When women make the decision to leave their homes and countries, the decision is not only economic, but also personal and emotional, especially when they leave children behind. This study examines one aspect of transnational migrant women's lives, that is, mothering from afar. This is an important social phenomenon to study, as it has implications for transnational family cohesion, childhood experiences, and motherhood.

While economic and exploitation aspects of migrant work (e.g., Ruzungunde and Zhou, 2021; Vosloo, 2020) and domestic work (e.g., Fenton and Fitchett, 2025; Maboyana and Sekaja, 2015) in South Africa have been widely studied, the aspect of transnational mothering by Zimbabwean migrants lacks scholarly attention.

Through their work, Madziva and Zontini (2012) demonstrate that it is almost impossible for Zimbabwean transnational mothers to provide for their children and maintain emotional intimacy due the strict immigration laws of the United Kingdom, highlighting the importance of family resilience specifically when it comes to guardians' ability to provide and care for the absent mothers' children. Maintaining emotional connections is not enough for these mothers, they must also send remittances and maintain ongoing communications to show their children and those caring for them they are still present in their absence (Madziva and Zontini, 2012). While transnational mothering often leaves the mother with feelings of failure brought on by the inability to pull their families out of poverty no matter how hard they try (Muñoz, 2019), other findings have challenged dominant narratives about women that leave their children behind. In a study of transnational mothers, their children and their children's caregivers, Takaindisa (2020) shows firstly that separation from family must happen and is for the good of the family as it allows breadwinning to take place; secondly that children are not to be perceived as just individuals in need of care but can themselves show up as necessary caregivers. Further, they can accept the absence of their mothers when they can see perceive this absence as instrumental for family survival; and thirdly that caregivers take care of the left-behind children expecting reciprocity in future. Caregivers also do not take on all the responsibilities of motherhood, leaving aspects such as discipline for their biological mothers.

The necessity of this study lies in adding to the dearth of existing body of knowledge on the mothering experiences of Zimbabwean domestic workers in South Africa. The studies that exist tend to focus on the experience of the mother, leaving the experiences of the child largely understudied (Asis and Feranil, 2020) although scholarship in this area is growing (Chingwe et al., 2025). This study contributes to the body of knowledge by exploring underrepresented emotional and relational elements of mothering across borders, by looking at the visitation and strategies of mothering left-behind children from a distance. The experiences of children are also considered in understanding transnational parenting.

This research sought to gain insight into how Zimbabwean domestic workers in South Africa navigate mothering while separated from their children. By conducting in-depth interviews with Zimbabwean mothers who have migrated to South Africa to perform domestic work, we interrogated the effects of prolonged separation on the part of both the mothers and their children.

We chose to focus on Zimbabwean migrant domestic workers for the following reasons. Firstly, there has been an increase in the number of Zimbabwean women who migrate to South Africa (Tame, 2018). Many of them find work as domestic workers because of the low barriers to access that are present in this sector (Tame, 2018). Owing to the nature of domestic work, there is generally little opportunity to visit their families who remain back home. These women work in a foreign country while simultaneously trying to maintain ties with their children who remain in Zimbabwe (Thebe and Maombera, 2019). Secondly, although the experiences of Zimbabwean women in South Africa have been documented in the literature, they have not been explored from the perspective of family separation and parenting across the border. By foregrounding the voices of Zimbabwean domestic workers, this paper contributes a contextually grounded understanding of transnational motherhood by revealing how structural constraints, financial constraints, and emotional burdens shape cross-border parental practices across the border.

## 2 Literature review and theoretical framing

### 2.1 South–south migration

Migration is a common human undertaking that is often driven by globalization, politics, and economics. South–South migration, the migration between the developing countries of the Global South (Bakewell et al., 2019), makes up more than one-third of global migrations (Schewel and Debray, 2024). It is propelled by factors such as proximity, informal labor markets, and intra-regional networks. According to the United Nations. (2019), more people move between developing countries than between developing and developed countries. Despite the scale thereof, South–South migration is still understudied. Some 80% of South–South migration takes place between countries that share borders (Shaw and Ratha, 2007). This is because such migration is less costly and the process is eased somewhat by ethnic and community ties with people from the same country and region. Of interest to this study is migration in sub-Saharan Africa, where political and economic strife drives movement within the region. Movement by migrants is often for informal labor purposes, including domestic work, a field dominated by women (International Labour Organization., 2021).

The major factor in the large-scale migration from Zimbabwe is political instability which gave way to major economic challenges (Vorvornator and Enaifoghe, 2024). Unemployment skyrocketed and the highly unfavorable economic conditions caused a surge in poverty levels (Madebwe and Madebwe, 2017; Van Klaveren et al., 2010). These factors diminished people's ability earn a living (Madebwe and Madebwe, 2017). The introduction of the Economic Structural Adjustment Programme (ESAP) in 1991 was one of the major causes of the exodus (Hadebe, 2022). Its failure has been a key contributor to Zimbabweans exiting their country to becoming labor supply to the care, agricultural, security, teaching and domestic work industries (Crush and Tevera, 2010).

Following Zimbabwe's multi-layered crises of the early 2000s, millions of Zimbabweans have been driven to pursue their livelihoods in other countries (Hammar et al., 2010), with or without documentation (Hansen, 2024). In keeping with the legacy of colonial-era labor migration routes, most have moved to South Africa (Musoni, 2020). With its relatively strong economy, South Africa is particularly attractive to Zimbabweans, as they perceive the country to hold better economic prospects for them (Landau and Segatti, 2009) until they may return home at the end of their working lives (Hansen, 2024).

The number of Zimbabwean women migrating to South Africa has been steadily increasing over the years (Bamu, 2018; Chisale and Gubba, 2018), and many of these women eventually find work as domestic workers in the country. Domestic work is a low-skill job that is popular among immigrants globally because of its low barrier to access (Tame, 2018). However, Chisale and Gubba (2018) argue that the commonly held belief that domestic work is performed only by uneducated women has been proven to be false, as increasingly, educated women are taking employment as domestic workers. In South Africa, the domestic work sector employs educated women migrants from Southern African Development Community (SADC) countries, including Zimbabwe (Chisale and Gubba, 2018).

The demand for domestic workers in South Africa has been on the rise since the late 1990s (Blackett, 2011). This increase in demand is argued to be due to the rise of dual-income households in which women have careers outside the home (Blackett, 2011; Du Toit, 2012). Although there is a large demand for domestic workers, the cost of employing a domestic worker in South Africa has increased since new labor regulations were introduced. Although these labor laws (e.g., the Basic Conditions of Employment Amendment Act, No. 7 of 2018, and the National Minimum Wage Act, No. 9 of 2018) were designed to protect domestic workers from exploitative labor practices, they may sometimes have unintended consequences. After the National Minimum Wage Act was introduced, local domestic workers became more expensive to hire at the legally mandated minimum wage (Nqambaza, 2016). Employers tend to prefer hiring migrant women to perform domestic work because they are willing to accept less pay (Albin and Mantouvalou, 2012; Motala, 2010) than locals for the same work. Unlike locals, migrant domestic workers who do not possess the appropriate permits are easily exploited and replaceable (Tame, 2018), since they do not enjoy the protection of South Africa's labor laws. These factors increased the demand for the cheaper labor of migrant domestic workers (Nqambaza, 2016).

### 2.2 Leaving home behind

Bamu (2018) notes that the high costs associated with moving to South Africa preclude Zimbabwean migrant domestic workers from bringing their spouses and children to South Africa. Many of these women are therefore compelled to make the choice to leave their families behind in Zimbabwe (Dinat and Peberdy, 2007; Filippa et al., 2013). This has emotional consequences for these women, which reinforces the need to understand emotion in the context of migration (Hoang and Yeoh, 2012; Tolla, 2013). Castañeda and Buck (2011) note that individuals who migrate to other countries are confronted with many psychological challenges. Several studies have found that immigrant women may experience post-traumatic stress, loneliness due to separation from family and friends, poor social and support networks, and may also suffer from low self-esteem (Hall et al., 2019).

For migrants who are mothers, leaving their children behind and living far away from them can be experienced as a daily emotional struggle and a highly stressful experience that influences their overall wellbeing (Schmalzbauer, 2004). In their study, Thela et al. (2017) found that leaving one's family behind is associated with depression, and they argue that within the context of the host country, family separation may be experienced through feelings of helplessness and hopelessness. This adjustment usually involves emotional disruptions and disconnections (Hoang and Yeoh, 2012) and may have a negative impact on the worker's emotional wellbeing (Tolla, 2013).

This is the case of these Zimbabwean women who have left their homes and families in Zimbabwe to work for and live with another family in their home. They must cope with the separation from family while finding a job, accommodation, possibly learning a new language and familiarizing themselves with the new culture.

Silver (2011) argues that being separated from family can induce stress and depression. These negative effects are generally more present in women because the family tends to play a more significant role in their lives (Silver, 2011). Regardless of how long family members have been separated, family members in both countries experience emotional strain as a result of being away from family, including feelings of worry, depression, loneliness, and loss (Solheim and Ballard, 2016). (Hall et al. 2019) reveal that female migrant domestic workers tend to also experience a decline in social networks and support. The separation of women migrants from their families can be a stress-inducing life event that causes a breakdown of support networks, which may make it more challenging to cope with everyday life stressors (Silver, 2011).

The children who are left behind may suffer negative consequences as a result of separation from their parent(s). These include feelings of loneliness and anxiety, which may have detrimental effects on the child's academic progress (Filippa et al., 2013). In pursuit of a better life for their children, migrant domestic workers spend long days at work caring for their employers' children, while only parenting their own children from a distance (Phillips, 2011).

Crush et al. (2015) maintain that in South Africa, Zimbabwean migrants generally remain in contact with their family left behind in Zimbabwe. Similarly, Dinat and Peberdy (2007) found that most migrant domestic workers in South Africa, particularly those from countries in the SADC region, preserve their relationships with those they leave behind in their home countries. How they manage to maintain communication over national boundaries, Hoang and Yeoh (2012) suggest, provides an interesting way of exploring how the transnational Zimbabwean–South African family maintains its intimacy and emotional ties. Another way of maintaining contact is by sending money and other material goods to their home countries.

### 2.3 Remittance-sending

Making a living is the reason for most transnational labor migration, as migrants want to be able to support the families they leave behind (Adem, 2021). Filippa et al. (2013) note that the decision to leave one's family, particularly children, to pursue work is made under the belief that the remittances they send home will provide those left behind with a better life. Research shows that Zimbabwean migrants living in South Africa often send remittances to their families in Zimbabwe (see Nzabamwita, 2018; Sithole, 2022). Remittances, in this context, refers to the money and/or resources that Zimbabwean migrant domestic workers send to their families in Zimbabwe during their stay in South Africa (Castañeda and Buck, 2011). Nzabamwita (2018) and Sithole et al. (2025) distinguish between formal and informal remittance-sending. Formal remittances are those sent through recognized financial institutions such as banks, whereas informal remittances are those sent using informal channels, such as sending cash with friends (Nzabamwita, 2018).

Remittance-sending by migrants points to strong ties between them and the family members back in Zimbabwe (Castañeda and Buck, 2011). Zimbabwean migrants commonly remit grocery staples such as grains (rice, maize meal, and cereals), dairy products, and perishable (fish and meat) and non-perishable foods (Sithole et al., 2025). Nzabamwita (2018) found that Zimbabweans tend to remit more than other African migrants in South Africa. Makina (2013) found that migrant women are generally more consistent than male migrants in the amounts they send back home and tend to remit a higher proportion of their salaries than men do. Younger migrants tend to remit lower amounts and less frequently than their older counterparts (Solidarity Peace Trust., 2009). Furthermore, Nzabamwita (2018) found that a migrant's income and level of education are strong determinants of the value of their remittances. Nzabamwita (2018) found that those with relatively better education and income tend to remit more money than those with little to no education and little income.

Bamu (2018) estimated that Zimbabwean migrant domestic workers in South Africa may remit over 50% of their wages to their families back home, despite their low income. Castañeda and Buck (2011) found that, in cases where migrants had not made enough earnings to be able to remit, many preferred to stop all communication with their families rather than return to Zimbabwe as a “failure” with no money or resources. According to The Solidarity Peace Trust (2009), the reality is that many migrants do not remit as expected by their families. This includes not sending money at all, sending at very infrequent intervals, bringing goods and money only when they return home for the holidays, or sending money when they first find work but stopping over time. There are several reasons that migrants might not remit goods and money back home, including not being able to secure stable income and developing unhealthy social habits (such as drinking) that take up most of their money.

### 2.4 Transnational mothering

When migration seems to be the only way to take care of their family, mothers may need to live apart from their children for long periods at a time (Castañeda and Buck, 2011; Dinat and Peberdy, 2007; Nawyn, 2010). It is a persisting phenomenon that has been documented extensively in the literature (see, e.g., Ballaret and Lanada, 2021; DePalma et al., 2022; Haagsman and Mazzucato, 2021; Khosravi Ooryad, 2022). Migrant domestic workers may be compelled to leave their children behind in their home country to be cared for by their spouses, mothers, sisters, or other family members, even extended family (Clarke, 2002; Suárez-Orozco et al., 2011). Hondagneu-Sotelo and Avila (1997) named this phenomenon “transnational mothering.” Migrant domestic workers who are also mothers do not give up their parental duties when they leave; however, they often find it challenging to maintain a bond with their children when they are away for a long time (Hoang and Yeoh, 2012; Suárez-Orozco et al., 2011). Migrant mothers are expected to maintain significant emotional ties with their children despite the distance between them (Dreby, 2006).

To cope with being away from their families, women who mother transnationally experience social and psychological strain, emotional distress, and at worst, depression (Ballaret and Lanada, 2021; Pineros-Leano et al., 2021). They turn to prayer, friends, optimism, and virtual communication to try and counteract these negative effects (Ballaret and Lanada, 2021). Mothers in this situation tend to re-evaluate their mothering and reconstruct financial support as a care (DePalma et al., 2022).

Understanding transnational motherhood involves looking at how parenting is practiced and experienced in the context of geographical separation (Carling et al., 2012). Baldassar et al. (2014) argue that the concept of transnational mothering challenges the dominant view about the bond that exists between mother and child and the implications associated with the mother and child's being physically distant from each other. Usually, when mothers migrate, relatives step in to care for the left-behind children (Carling et al., 2012). Baldassar et al. (2014) argue that transnational families continue to maintain their bonds even though they may not be physically present in each other's daily lives.

Children also feel the impact of the mother's absence. Parental migration has left an increased population of children to be cared for by other family members (Chingwe et al., 2025). Since most studies on transnational families tend to focus on mothers (Asis and Feranil, 2020). Chingwe et al. (2025) found that children left behind experience negative psychological, educational, economic and physical effects. Psychologically, their experiences are marked by hopelessness, misery, suicidal thoughts, rage and apprehension (Chingwe and Chakanyuka, 2023). Others also felt a sense of rejection and presented with symptoms of depression (Filippa, 2011). They can also be stigmatized, bullied and abused and vulnerable to abuse with no limited social support and protection (Tawodzera and Themane, 2019). According to Chingwe and Chakanyuka (2023), the psychological impact frequently leads to undesirable vices such as such smoking and drinking as well as violence and crime. Because some children have to take up the role left by their migrant families, these added responsibilities tend to create such a workload burden that they are not always able to meet the demands of school (Chingwe et al., 2025).

Despite the overwhelming negative effects, there are instances where the left-behind children thrive and these tend to be those with healthy coping mechanisms such as extra-curricula activities, participating in social activities (Makondo, 2022). In rare instances, remittances made provision for their educational needs thereby increasing positive outcomes for the children (Ndlovu and Tigere, 2018).

### 2.5 Experiences of Zimbabwean migrant women

Various researchers have documented the experiences of Zimbabwean female migrants in South Africa. Baison (2021) examined the recruitment and job-seeking strategies utilized by Zimbabwean women care workers in the domestic services sector. The findings of the study pointed to challenges in obtaining valid work visas and thus could not seek employment through formal channels such as recruitment agencies; they therefore favored social networks instead. Hlatshwayo (2019) studied the problems faced by Zimbabwean women, many of whom were domestic workers, when they traveled to South Africa in search of work. He found that women faced the double jeopardy of violence and lack of basic services and goods to maintain basic female hygiene. These women often employed individualized and isolated strategies to deal with these challenges. Makandwa and Vearey (2017) sought to understand the maternal health experiences of Zimbabwean migrant women in inner-city Johannesburg and found that their healthcare-seeking experiences were marred by verbal abuse and delays in receiving medical attention, especially at the critical time of giving birth. Ncube and Bahta (2022) wanted to understand how Zimbabwean migrant women adapted and coped while in South Africa. Their study revealed that human livelihood capital helped them navigate their environment. They also found that the women capitalized on South Africa's equality policies, which helped them survive. In their exploration of the feminized poverty that makes Zimbabwean women leave their home country and the sexual and gendered livelihoods that emerge as part of their survival strategies in South Africa, Batisai and Manjowo (2020) found that sexuality and migrations shape each other—an intersection that has not received academic attention. Their participants' experiences demonstrated that the subsumed sexuality in migration studies adds vulnerability to the Zimbabwean migrant woman identity. Chinyakata et al. (2019) aimed to understand the vulnerability of young female Zimbabwean migrants in Johannesburg, South Africa. The results pointed to the young women's being at risk of abuse (including from their intimate partners), discrimination, xenophobia, and economic marginalization in the workplace, at the hands of authorities, and from society at large. Lastly, Vanyoro (2019), in his study exploring the activism of Zimbabwean migrant workers in South Africa's domestic service sector, highlighted the importance of considering the intersectionality of nationality alongside race, class, and gender when dealing with the challenges faced by this population.

Of the existing research, none has explored the lived experiences of Zimbabwean women domestic workers who leave their families behind to settle in South Africa. Against this background, in this study we focused on the lived experiences of such women. Our aim was to develop an in-depth understanding of how Zimbabwean migrant domestic workers in South Africa experience family separation and transnational mothering. With this research we therefore seek to contribute to the body of literature concerned with migrant domestic work by examining how Zimbabwean migrant domestic workers in South Africa experience family separation.

### 2.6 Theory of ambiguous loss

This study, primarily grounded in the experiences of migrant domestic worker mothers, is informed by the theory of ambiguous loss (Boss, 1999). Ambiguous loss is experienced when, for the individual, the loss is unclear in terms of how long it is going to last or whether a loss has even occurred Huebner et al., (2007). The uncertainty has much to do with the fact that ambiguous losses are not as easily identifiable as other losses such as death (Betz and Thorngren, 2006). However, (Huebner et al. 2007) note that, although people may experience much uncertainty in such a situation, they are different in how they process that uncertainty, therefore resulting in different experiences of loss, which can either be physical or psychological (Betz and Thorngren, 2006). Individuals also differ in how they are able to cope with ambiguous loss, as some are more resilient than others (Pérez, 2015). De Leon (2005) argues that immigrants may suffer from ambiguous loss. Boss (1999) theory of ambiguous loss therefore offers a useful framework for exploring how migrants experience and cope with separation from family. Boss identifies two types of ambiguous loss: the first is characterized by a physical absence of the individual while maintaining their psychological presence, and the second occurs when the individual is present physically but psychologically absent. The first is relevant to this study. When individuals are separated from their family across national borders, they may experience ambiguous loss (Juabsamai and Taylor, 2018; Mazzarelli et al., 2021). The mother leaves home, but she remains in the minds of the family left behind and vice versa. Ambiguous loss has psychological and psychosocial implications for the migrant and her family and for social and community contexts (Mazzarelli et al., 2021). Consequently, in order to make sense of the ambiguous loss, it is important to understand the ethnic and cultural contexts in which the loss occurs (Boss et al., 2003).

Solheim and Ballard (2016) argue that those immigrants who migrate independently, leaving their families behind, often experience greater levels of stress than those who travel with their families. In such cases, family members in either country can experience grief and loss in their separation. They argue that the ambiguous loss resulting from transnational family separation (when families are separated across national borders but remain psychologically present in each other's lives) may lead to a blurring of boundaries. A migrant may experience ambiguous loss when their family is still alive but far away and they do not have access to them or cannot reach them at will (De Leon, 2005). Unlike the finality of death, in the case of migration, there is always the possibility of dreaming about the return of those who are gone so that they may reconcile with their family. The limited global scholarship on migration and ambiguous loss suggests that when a parent is separated from their children, the parent tries to make up for their physical absence by maintaining their psychological presence through sending remittances or frequent communication (Juabsamai and Taylor, 2018).

Against the background set out in the preceding sections, the central research question for this study was: how do Zimbabwean domestic workers in South Africa experience and navigate transnational motherhood? Specifically:

- What are the constraints associated with visiting home?
- What emotional difficulties do migrant mothers experience when leaving their families behind?
- How does the migration of their mothers affect the left-behind children?
- What are the challenges that migrant mothers face when sending money home?

### 2.7 Positionality

In addition to the standard ethical considerations, we evaluated our positionality and the implications it had for our study into a sensitive topic.

The first author was a master's student at the time of data collection. She is a young Black South African woman who recognizes that she holds a complex position in the context of this study. There was racial proximity to the participants which may have helped elicit trust, candor and shared understanding during the interview. Simultaneously, her South African citizenship situates her differently from the participants who hold a marginalized identity as migrant women and face realities she cannot fully comprehend as transnational mothers. Due to her role as researcher, she may be perceived by the participants as a figure of authority, which made it important for her to be aware of the power imbalances and emphasize empathy, respect and their position as experts in their own experiences.

The second author is an academic and supervised the first author during her master's studies. One of her key research focus areas is the Black condition and understanding the experiences of Black people from a social and work perspective. Therefore, this study was of interest to her. Although she is an African mother herself, she came into the study acknowledging her privileged position as a middle-class individual and as an outsider, without first-hand experience or deep understanding of what it means to leave one's children behind out of necessity. Of great importance to her when conducting and supervising this study was the fact that she employs a migrant domestic worker, without whom she could never do what she does. She believes that understanding migrant domestic workers' struggles allows her and others not to become part of their ongoing challenges but to be co-seekers of solutions.

## 3 Research method

In order to fulfill the research objectives and answer these questions, we adopted a qualitative approach, grounded in a constructivist-interpretivist paradigm. The qualitative approach was suitable in this case because we were concerned with both meaning and context, that is, the meanings that Zimbabwean domestic workers create around their mothering experiences while in South Africa.

We employed purposive sampling to recruit only those participants who had experience of the phenomenon being studied. The study participants had to meet the following inclusion criteria: (a) be a Zimbabwean woman migrant currently working as a domestic worker in South Africa, (b) have been living in South Africa for at least 6 months, and (c) have children back home in Zimbabwe. Additionally, they had to be able to communicate in English. Since we anticipated that the population of Zimbabwean migrant domestic workers might be difficult to find (which proved to be the case), we recruited additional participants using snowball sampling. The first participant put the first author in touch with some of her friends from church who met the selection criteria. No new participants were recruited after we reached data saturation at nine participants.

A summary of the participants' biographical details is given in Table 1.

**Table 1:** Demographics of participants.

**Pseudonym**	**Age**	**Length of time in South Africa**	**Family left behind**
Rudo	35	5 years	Child (7), sister, nieces, and nephews
Tsitsi	37	7 years	Son (16)
Tarisai	45	5 years	Sons (16; twins, 24)
Tatenda	32	4 years	Son (5), daughter (7), mother
Chiedza	39	6 years	Son (9), mother, father, sister
Gamuchirai	58	22 years	Daughter (31), grandson (14)
Rutendo	36	7 years	Daughter (15)
Fadzai	60	17 years	Children, brothers, sisters
Chido	58	19 years	Children, parents

Semi-structured interviews are one of the most widely used methods of gathering data in South African migration research (see Kanayo and Anjofui, 2020; Masebo, 2018). Through face-to-face semi-structured interviews, the first author was able to directly access the experiences of participants and probe for clarifications, examples, and elaborations as needed. Examples of questions and prompts include “How do your kids back home cope with your absence for the greater part of the year?” and “Tell me about your visits home.” All of the interviews were conducted in English.

Both authors analyzed the data, using Braun's and Clarke (2006) thematic analysis technique. This allowed us to identify, analyze, and report themes and capture nuances and complexities in the data. The first author transcribed the interviews verbatim and performed a quality check on the transcripts. We performed open coding on the first two transcripts together. This was done as a way for the second author to train the first author on how to complete the process. For the rest of the transcripts, the second author played a guidance role. She quality checked the final product. Then during manuscript preparation, the second author reviewed and refined the coding framework. Both authors were responsible for ensuring that the themes and subthemes were reflective of the data and research questions. The analysis involved open coding to identify and code data that helped answer the research questions; sorting the codes and grouping like codes; generating subthemes and themes; reviewing the themes to ensure each code was housed under the correct theme and that each theme was sufficiently different from the others; naming the themes; and finally, compiling a report of the findings.

Ethical clearance was granted before data collection commenced. The participants took part in the study voluntarily. They were aware that they could withdraw at any time from the study, without adverse consequences or having to explain themselves. Before the interviews took place, each participant consented by signing a consent form. As we believed that in talking about their experiences the participants might touch on issues of a sensitive nature that could cause them distress, the first author informed the participants that if they shared sensitive information about themselves that led to negative emotional consequences, they would be referred to an experienced counseling psychologist at no cost to them. The participants shared without any perceivable hesitation or discomfort. The data was stored on the password-protected laptops of the researchers, and the participants were made anonymous through the use of pseudonyms.

## 4 Findings

The analysis yielded three separate but intertwined themes around the participants' transnational mothering experiences.

### 4.1 Theme 1: infrequent homecoming shaped by structural barriers

This theme encapsulates the participants' experiences of their visits home to their families in Zimbabwe. The participants all expressed that, although they could make the trip home, it was rarely more than once a year and the visit only lasted a few weeks. These holidays were in December–January and coincided with the festive season, when families in South Africa (their employers) would be on leave.

“*Three years ago I was visiting [Zimbabwe]. I just to go there to stay for three weeks then I came back.” (Tatenda)*

“I always visit during December on the 20th. Every year I will be going on the 20th [of December] and coming [back] on the 15th January. Then I will spend the whole year here not seeing my family.” (Tarisai)

The participants felt that this was not enough, as they missed their homes and their children. However, going home is expensive, which limits the frequency of the visits.

“If you don't see your child maybe for a year you only see your child once in December, it's so painful. Because you can't go always. You must budget.” (Tsitsi)

“Some years I go three times a year and some years when I'm financially constrained, I only go once at the end of the year in December.” (Fadzai)

The participants lamented that, in addition to limited funds, their work requirements were another impediment to their going home—the families they work for need them around all year round and allow for December holiday visits, whose length is determined by the employer's needs.

“At home, I don't stay for a very long time. It's like I stay according to my boss, how long did she give me. If she say two weeks, it's two weeks, I come back. If she say it's a month, it's a month, I come back.” (Rudo)

The participants in the present study faced challenges visiting home, including limited finances and leave. There is limited literature on Zimbabwean migrant workers' visiting home, but a few issues can be highlighted from what is available. The above stories from the participants reflect brief and sporadic reunions with family at home, indicating prolonged absence from family which may contribute to unresolved longing. This is indicative of ambiguous loss (Boss, 1999). Their ability to visit mean that they are never fully present, yet never fully absent.

Thebe and Maombera (2019) note that one of the key reasons for limited visitation for domestic workers is employment terms, which suggests that some domestic workers receive less leave than is due to them by law. The time off given to the domestic workers in the present study was not abnormal, however, as they seemed to receive the same number of leave days as South African workers—or more in some cases. According to the Basic Conditions of Employment Act (No. 75 of 1997), employees are entitled to 21 consecutive days of annual leave. One of the participants spoke of taking only as much time as was given to her by her employer, which alludes to the employer–employee power dynamics that must be negotiated. Due to their vulnerability in this relationship, the domestic workers rely on the goodwill of their employers to determine meaningful and just time off, which should be guided by the Basic Conditions of Employment Act but is often not. A further challenge for migrant workers is that they do not live with or near their families, so the time off seems insufficient (compared with local workers) when they have not seen their families for prolonged periods of time. Furthermore, although this was not mentioned by the participants in our study, since many domestic workers work under exploitative conditions (Nayupe et al., 2023), their freedom to travel may be severely restricted in many cases, which may hamper their ability to maintain bonds with family members.

Financial constraints hindered the participants' ability to visit Zimbabwe because they send much of their income back home through food and monetary remittances, thus limiting their ability to save money to travel back themselves. This finding aligns with those of Namuleme (2019) and Zikhali (2016), who contend that women migrants employed in low-wage jobs such as domestic work are often pressured to be separated from their families for extended periods of time due to financial constraints.

Although we did not ask the participants their legal status, literature points to nuances in the ability of migrants to visit home. For example, if one works in South Africa illegally, this significantly restricts one's movement across borders (Moyo, 2019). Should they want to cross illegally, they need to pay to do so (Moyo, 2016).

### 4.2 Theme 2: vulnerabilities and unseen challenges of children left behind

This theme captures the experiences of migrant domestic workers' children living in Zimbabwe, through the lens of their mothers, the participants in this study. It describes how having to leave one's children leads to poorer outcomes for them.

Tsitsi detailed how, when she was living with her child, she ensured that scholarly matters were attended to, and her son showed strong academic potential. In her physical absence, her child's academic performance seemed to have declined, and she could not get to the root cause of the issue due to her being far away.

“My child is suffering. Sometimes— Long back my child was very bright at school but now he's going down down down, which I don't know what's going on.” (Tsitsi)

Furthermore, the children miss their mothers and long for them to be together, which is almost always impossible. Ironically, mothers are separated from their children for the good of their children. Tsitsi relayed the story:

“He always told me that, ‘Ey, I wish, mama, I was there with you.' … I can't just come and stay with you while I'm doing something for you again, you see.” (Tsitsi)

Tsitsi also reported that migrant workers' children such as hers are mistreated and bullied by other children in the village. She spoke about how some other children took her son's clothes without asking while he was away at school. Her son also reported that another child in the family had beaten and hurt him badly. Both she and her son were powerless to stop the abuses from the other children, and this made her wish that he lived with her instead, so that he did not have to experience these abuses, which caused her distress, in turn.

“[My son] tells me, ‘When you are buying me clothes, they take my clothes.' … He's 16 years [old] and he's tall, and you'll find that some guys [who] are not going to school; when he's not around, he's at school, they took the clothes and [wore] them. … Last time he was beaten by the other child, by my cousin's child; he beat him badly. I was hurt. He was beaten the whole body. You know that stick which AmaZulu [the Zulus] they are using, that one?” (Tsitsi)

Tsitsi narrated yet another story about her son's being taken advantage of in her absence, this time by the adults who should have been caring for him. He sometimes went without food, even though she made provision for him. She was left to conclude that his caregivers did not like him, and she wondered whether her son received enough love from those around him. All of this was hurtful to Tsitsi.

“At times he will be telling me that, ‘sometimes I go to school, I do not eat' [‘I go to school without having eaten'] but it's so painful because you are sending money for food. The people who are taking care of him [don't] like him, you see. You see, it's hurting, so in time you realize that, eish, maybe my child doesn't find enough love.” (Tsitsi)

Tarisai believed that her absence might lead to her child's falling into destructive behavior and she would be none the wiser. Tarisai reasoned that, as she could not parent him meaningfully and provide him with the necessary guidance to make healthy choices in his teenage years, he might pick up unhealthy lifestyle habits such as smoking.

“If a child doesn't have motherly care, he will be like someone who doesn't even have someone who can tell him that ‘that thing which you are doing, this is bad.' No one can tell him. He can even smoke, if I'm there he can't smoke, you see.” (Tarisai)

Nonetheless, this did not deter Tarisai from trying to parent and make the effort to talk to her son to advise him to spend the money she sends responsibly. However, she conceded that she may never know the truth, as he would simply hide any bad habits from her during her short visits.

“Every month-end when I send him money, I will talk to him. … [My son] respects me very much but a child can even hide the things he does when you are not there. But when I'm there he respects me.” (Tarisai)

In contrast to Tsitsi and Tarisai's distressing accounts, Rudo reported that her child's experiences were positive, and as a result, she had fewer worries about being away from her child. She was happy about the treatment that her daughter received from her caregivers.

“I'm very happy because when I go there I just see they are living with [her] in a good way. … But if you just see, you see she gets enough food each and every time, she bath each and every time, so you just see she's living proper way.” (Rudo)

These findings echo those of Zikhali (2016), who noted that Zimbabwean migrants often perceive their children to be better off when left in the care of female relatives such as aunts and grandmothers. The study participants' accounts suggest that, although Zimbabwean transnational mothers leave their children in the care of relatives in Zimbabwe, hoping they will be safe and well taken care of, this is often not the reality. While many of the participants in the study expressed satisfaction with the caregiving arrangements they have organized for their children, one reported neglect. According to Asis (2006), when the children receive the required care, their wellbeing is maintained and the void left by the transnational parents is filled as best as possible.

However, the separation carries some risk, as it may expose children to dangers that parents (mothers, in the case of this study) have no sight of or control over (Haagsman, 2018; Larrinaga-Bidegain et al., 2024). This becomes a source of anxiety for mothers, as some of the domestic workers in this study recounted. To try to overcome this anxiety and monitor their children's wellbeing, they maintain regular communication with their children, which also allows them to provide advice, encouragement, and affirmation, as Namuleme (2019) and Thebe and Maombera (2019) found. However, despite these efforts, they worry that their children might associate with negative peer groups and adopt high-risk behaviors. Chib et al. (2014) reported a similar finding in their study: transnational mothers often worried that their children might engage in drug or alcohol abuse and perform poorly in school. The moms feel guilty about this and often blame their absence for the change in their children's behaviors, a pattern also observed by Lockwood et al. (2019).

The term “diaspora orphans,” a term now part of the Zimbabwean public discourse, describes children whose parents have migrated to another country but left their children behind (Kufakurinani et al., 2014). They have been stereotyped as delinquent, emotionally neglected, and lacking structure. According to Kufakurinani et al. (2014), global studies show that such children often develop depression, anxiety, and resentment toward their parents. A Mexican study demonstrated how the children of migrant workers had fewer educational aspirations and were more likely to drop out of school (Dreby, 2007; Zentgraf and Chinchilla, 2012).

Although a plethora of literature acknowledges the trauma of parent–child separation, problematic assumptions underlie this discourse. The psychological damage is evaluated through a Western lens that prioritizes the Western nuclear family and model of attachments and often disregards culturally relevant models (Mazzucato and Schans, 2011). In Africa, extended family caregiving is common and socially accepted; therefore, the same assumptions cannot be made in this context, and a culturally sensitive understanding of transnational mothers and their children is needed for a more nuanced understanding of the matter (Kufakurinani et al., 2014).

### 4.3 Theme 3: transnational mothering

In exploring how the participants maintained links with those they left behind in Zimbabwe, the theme of transnational mothering emerged. The women in the study, although separated from their children, tried to live up to their role as mothers. This theme, comprising three subthemes, details how the participants experienced mothering across national borders.

#### 4.3.1 Subtheme 1: longing and emotional labor of absence

A very common theme among the women was missing their children, parents, and Zimbabwe as home, an expected phenomenon given ties with family members and the long periods without seeing them. Many of the participants reported feeling these emotions continually.

“I was thinking of [my child and grandchild] them every day. I was thinking of them.” (Gamuchirai)

“I took it one step at a time but it was so hard because I would think of my parents every day, I would think of my kids every day.” (Chido)

The moments of missing family back home did not start after long periods of not seeing them, but almost immediately, at the moment of separation, such as on the bus to South Africa, as Tatenda described. Given the extended period of separation and sometimes not knowing how long it would be until they saw their families again, it was a painful experience for the mothers. Although she may be physically in South Africa, a mother's heart is at home with her children, as Rudo put it.

“My heart it was so painful. I remember the time I was crying in the bus. Yes, then I was just— The bus was standing to go in the road. I just, eish— [pause] My mind was still thinking about my kids, yoh.” (Tatenda)

“I'm here but my mind and my spirit is at home.” (Rudo)

The parents in this study expressed missing their parents and children constantly. Our findings reflect what we already know about transnational mothering. Zimbabwean mothers navigate the complex web of diasporic responsibilities of having to meet economic needs, mother from afar, and live through the separation from their children. Madziva and Zontini (2012) state that prolonged separation from their children is emotionally taxing on mothers, and at times even ambivalent: the migrant mothers balance the necessity of being away from their children to provide financially for them with the emotional bond that makes them want to be with their children physically. This is true for Zimbabwean mothers who seek opportunities in other countries in the hopes of securing better outcomes for their children (McGregor, 2007). These families rely on phones to keep up communication, which can help mitigate the feelings of absence and maintain familial bonds despite physical separation (Madianou, 2012). The use of such technologies allows mothers to participate in parenting practices that reinforce their maternal roles, even from afar.

#### 4.3.2 Subtheme 2: financial remittances and extended obligations

All of the participants shared that their primary reason for migrating to South Africa was to find employment so that they could provide for their children. However, their children are not the only ones that benefit from this money. Caregivers such as the children's grandparents and aunts also receive a share (in addition to the money to cover the children's needs) as gratitude for taking care of the children. Importantly, since the people that immigrant workers leave behind often live in poverty, there is the expectation that such workers will help their family when required. Some of the participants listed their obligations as follows:

“I send money at home, like [to] my parents, every month. Sometimes at home groceries are too expensive; I have to buy grocery here and send at home to my parents, even to my baby. She's at school but I have also to buy the grocery for her. On top of that, I have many people to take care of.” (Rutendo)

“I had to take some of my salary and send it to [my son] in Zim. Not to him only, but to my parents as well because no one was taking care of my parents. There's a certain amount that I take home to help there because I've got my aunts there to help at home.” (Chido)

At times, however, those back home must face disappointment from their migrant relatives, since they do not always have the money to meet the demands at home. This is especially true for those who are not employed full-time and therefore have no consistent income. For example, Tsitsi's extended family, who were looking after her son, would sometimes demand more than she had to give. She expressed:

“Once you don't send, yoh, they will call you and tell you something else … and even if you are sending they will still complain that the money that you are sending is too little. And even if you're telling them, ‘these days I'm not working.”' (Tsitsi)

Similarly, Tatenda shared:

“For now I just [am able] to pay the rent and buy the food [for myself in South Africa]... I don't have the money to send for the home [in Zimbabwe].” (Tatenda)

Fortunately, her family was more understanding than others, and her sister would step in and provide for her children:

“Sometimes it's my sister [who helps] my kids.” (Tatenda)

Existing literature (e.g., Sithole et al., 2025) clearly describes the difficulties of having to sustain oneself as a Zimbabwean migrant in South Africa while having to send money back home. Remittances are an important source of financial support, especially given the economic hardships in Zimbabwe. For our participants this was also the case. They supported not just their children but their families too, which are broad, by definition in the African sense. However, to our knowledge, no studies detail the dynamics of migrants' depending on family members to use their remittances to pay for necessary expenses in their absence but also having to contend with the misspending of the same.

#### 4.3.3 Subtheme 3: fractured bonds and emotional withdrawal

Having left her daughter in Zimbabwe 22 years before, Gamuchirai believed that their mother–daughter bond was somewhat fractured because they did not communicate as openly as she wished:

“There are some things she doesn't tell me.” (Gamuchirai)

Similarly, Tarisai believed that in her absence, her sons' behaviors have changed, pointing to how their relationship would have been closer had she been able to be in close proximity to her son while he was growing up:

“The way I used to be with them and the way they are acting when we are separated, it's different. As a mother, you can see that it is different. It's not the way they were doing when I started having them.” (Tarisai)

Tsitsi relayed that a similar distance had formed between herself and her son. Instead of being excited to hear his mother's voice on the phone, he had become withdrawn.

“The situation is not that good. So, you find that sometimes you call him he won't say much.” (Tsitsi)

The transnational mothers in this study articulated the distress they experienced in being separated from their children. Adem's (2021) study on transnational families revealed similar findings and suggested that the physical distance created between mothers and their children generated psychological strain for them, a hardship that is structurally imposed and not voluntarily chosen. While migrant mothers face many challenges, sustaining maternal intimacy is chief and most precarious among these (Baldassar, 2008; Ryan, 2008). The accounts of the participants in this study point to the emotional ambiguities that migrant mothers must navigate. Through Boss's ambiguous loss lens (Boss, 1999; Boss et al., 2003), we understand that mothers experience a loss that they cannot clearly define in being separated from their children but remaining invested by calling and caring for them from a distance. It is a loss nonetheless, as the mother is simultaneously absent and present, thereby creating a continual sense of emotional strain.

### 4.4 Implications

This study extends the application of Boss (1999) Theory of Ambiguous Loss into the Southern African context, particularly the experiences of transnational Zimbabwean domestic worker mothers living and working in South Africa whilst leaving their children back home. While one of the ways in which ambiguous loss is positioned is psychological presence despite physical absence (Boss et al., 2003), our study suggests that this psychological presence is not guaranteed and is often neither stable nor secure. While a participant was satisfied with the care received by her children, in other instances, prolonged separation often led to emotional distancing by the children, strained communication and uncertainty about the state of the children's emotional states, reflecting the blurring of boundaries and unresolved grief that Boss (1999) refers to in her conceptualization of ambiguous loss. This lack of uniformity in experiences challenges the assumption that ambiguous loss leads to emotional distress. This emotional tax may be mediated by the quality of care received by children at home, social support by family and access to communication (see Chib et al., 2014; Madianou, 2012). Future scholarship in migration should pay close attention to this aspect.

South-South migration presents us with ambiguous loss dimensions that are under-researched. Participants described the emotional toll of having to fulfill family obligations to children and other family members whilst simultaneously having to see these remittances misused. These findings align with Castañeda and Buck (2011) and Adem (2021) but add culture-specific insight, that psychological presence can not only be maintained through emotional connections, but also through financial provision that does not always meet the needs of the child. This expands Boss (1999) framework to include financial visibility as a means of maintaining presence, over and above physical and psychological.

Our findings challenge dominant, western-centric discourses that pathologize transnational parenting (Kufakurinani et al., 2014; Mazzucato and Schans, 2011). In African contexts, child-rearing is a communal and involves extended kin networks. Therefore, absence due to migration does not automatically signal deficiency in parenting. Therefore, an African lens must be applied to African family and mothering practices fully understand the meanings of transnational mothering in context. However, even with these networks in place, domestic workers often experience difficulty in intervening in challenges faced by their children from afar, thereby exacerbating ambiguity and helplessness in the loss. Our study both supports the theoretical assumptions of ambiguous loss (Boss, 1999) while simultaneously adding a new lens of how we understand it indicating that ambiguous loss is shaped by relational, socio-economic and cultural structures.

There are implications for domestic workers who mother transnationally. Firstly, the mothers who participated in this study relayed stories of how they were affected by being separated from their children due to socio-economic circumstances. The separation affected them psychologically, as evidenced by their mentioning the pain they felt. Transnational mothers may expect to experience emotions of guilt as a result of the separation and time lost, anxiety and worry about the wellbeing of their children, and powerlessness to intervene in a meaningful way in their children's struggles back home.

Although transnational mothers cannot be with their children as often or for as long as they would like, they may find or form social networks with other domestic workers who are mothers and share the same struggle of being away from their children. This can provide some social support for them and alleviate the psychological burden of being away from their families. Given the number of Zimbabwean women working as domestic workers in South Africa, there should be cooperative savings groups or group savings clubs to help the women save for home visits and emergencies. The South African “stokvel” model could be used: members contribute an agreed-upon amount each month, from which a lump sum is paid out to each member, in turn.

### 4.5 Limitations

The first author was acutely aware of the power dynamics between the participants—Black immigrant women—and herself—a South African who, although also a Black woman, held more privilege by virtue of her citizenship and socio-economic standing. Many of the interviews took place during a time when there was a spike in xenophobic attacks in the country, and she feared that the participants might assume that she herself was xenophobic. Many of the women she approached plainly refused to participate in the study out of fear, and so, with every participant who did agree to take part in the interviews, she made sure to cultivate trust by reiterating who she was, by stating that the participant's data was being collected for research purposes, and by assuring the participant of her anonymity. She checked in every so often with the participants during the interviews to ensure that they were comfortable continuing. The researcher came to the interviews as an “unknower,” with a stance of readiness to listen so that she might learn what the participants had to share, rather than assuming (Thomas and Sohn, 2023).

Appropriate and matched the interview questions.

### 4.6 Recommendations for future studies

This study offers an understanding of the transnational mothering experiences of Zimbabwean migrant domestic workers in South Africa. The findings offer a foundation upon which future studies could build. Firstly, the participants spoke of how family members were the ones caring for their young ones in their absence. The experiences for the children were mixed, with some having positive and others negative experiences. This phenomenon of children being cared for by grandmothers and aunts and how this impacts the children, mothers and caregivers, warrants more examination than currently exists. Secondly, when the domestic workers are far from home, they have no choice but to keep contact and “mother by telephone” through calls and WhatsApp. The nature of these conversations and how this impacts their relationships needs to be explored.

## Data Availability

The raw data supporting the conclusions of this article will be made available by the authors, without undue reservation.
